# The correlation between posterior tibial slope and dynamic anterior tibial translation and dynamic range of tibial rotation

**DOI:** 10.1186/s40634-021-00389-0

**Published:** 2021-09-02

**Authors:** M.J.M Zee, M.N.J Keizer, L Dijkerman, J.J.A.M van Raaij, J.M. Hijmans, R.L. Diercks

**Affiliations:** 1 Department of Orthopaedic Surgery, University Medical Center Groningen, PO Box 30.001, 9700 RM Groningen, The Netherlands; 2 Department of Human Movement Science, University Medical Center Groningen, University of Groningen, Groningen, The Netherlands; 3 Department of Orthopaedic Surgery, Martini Hospital, Groningen, The Netherlands; 4 Department of Rehabilitation Medicine, University Medical Center Groningen, Groningen, The Netherlands

**Keywords:** Anterior Cruciate Ligament (ACL), ACL reconstruction, Tibial rotation, Anterior tibial translation, Posterior tibial slope

## Abstract

**Purpose:**

The amount of passive anterior tibial translation (ATT) is known to be correlated to the amount of posterior tibial slope (PTS) in both anterior cruciate ligament-deficient and reconstructed knees. Slope-altering osteotomies are advised when graft failure after anterior cruciate ligament (ACL) reconstruction occurs in the presence of high PTS. This recommendation is based on studies neglecting the influence of muscle activation. On the other hand, if dynamic range of tibial rotation (rTR) is related to the amount of PTS, a “simple” anterior closing-wedge osteotomy might not be sufficient to control for tibial rotation. The purpose of this study was to evaluate the correlation between the amount of PTS and dynamic ATT and tibial rotation during high demanding activities, both before and after ACL reconstruction. We hypothesized that both ATT and rTR are strongly correlated to the amount of PTS.

**Methods:**

Ten subjects were studied both within three months after ACL injury and one year after ACL reconstruction. Dynamic ATT and dynamic rTR were measured using a motion-capture system during level walking, during a single-leg hop for distance and during a side jump. Both medial and lateral PTS were measured on MRI. A difference between medial and lateral PTS was calculated and referred to as Δ PTS. Spearman’s correlation coefficients were calculated for the correlation between medial PTS, lateral PTS and Δ PTS and ATT and between medial PTS, lateral PTS and Δ PTS and rTR.

**Results:**

Little (if any) to weak correlations were found between medial, lateral and Δ PTS and dynamic ATT both before and after ACL reconstruction. On the other hand, a moderate-to-strong correlation was found between medial PTS, lateral PTS and Δ PTS and dynamic rTR one year after ACL reconstruction.

**Conclusion:**

During high-demand tasks, dynamic ATT is not correlated to PTS. A compensation mechanism may be responsible for the difference between passive and dynamic ATT in terms of the correlation to PTS. A moderate-to-strong correlation between amount of PTS and rTR indicates that such a compensation mechanism may fall short in correcting for rTR. These findings warrant prudence in the use of a pure anterior closing wedge osteotomy in ACL reconstruction.

**Trial registration:**

Netherlands Trial Register, Trial 7686. Registered 16 April 2016—Retrospectively registered.

**Level of evidence:**

Level 2, prospective cohort study

## Background

Risk factors for ACL injury are multifactorial and, next to gender-related, genetic, and hormonal factors, include anatomical and biomechanical factors [14, 25]. One anatomical factor that has been of interest in recent studies is the amount of posterior tibial slope (PTS). From cadaveric experiments it is known that increased PTS leads to more forward-directed forces on the tibia and increases strain on the ACL [3]. Dejour and Bonnin showed that every increase of 10° in PTS leads to a 6 mm increment of passive anterior tibial translation (ATT) in ACL deficiency [10]. More recent studies confirm the correlation between PTS and passive ATT in both ACL-deficient and ACL-intact knees [8, 9, 13].

Increased PTS is related to increased risk of primary ACL injury and increased risk of graft failure after ACL reconstruction [6, 30, 32]. For this reason it has been suggested that, in revision cases, altering the amount of PTS by an anterior closing-wedge osteotomy could reduce strain on the ACL graft and prevent another re-injury [17]. It should be noted that past studies have evaluated passive ATT either using instrumented Lachman or in a cadaveric setting, both of which eliminate muscle tone. The influence of PTS on dynamic ATT is less extensively studied.

As clearly as the relation between PTS and passive ATT is demonstrated, less is known about the relation between PTS and tibial rotation. The ACL is known to restrict ATT, but also plays a role in limiting tibial rotation [12]. Due to the anatomical features of the tibial plateau, axial load transfers into an anteriorly directed force on the tibia [10]. This force increases with PTS [10]. As the medial and lateral tibial plateaus differ in congruency with the femur, as well as in mobility, we argue that the translation in the lateral compartment is more susceptible to changes in PTS. Due to this difference between the medial and the lateral compartment, axial load would not only be transferred into ATT, but also into rotation of the tibia relative to the femur. We hypothesized that this difference (referred to as ΔPTS) might be of more importance than the actual amount of slope itself, with respect to rotation.

If the range of tibial rotation (rTR) is related to the amount of PTS, a “simple” anterior closing-wedge osteotomy might not be sufficient to control for tibial rotation.

The aim of this study was to answer the following research questions:

- Is PTS correlated to dynamic ATT before and after ACL reconstruction?
- Is ΔPTS correlated to rTR before and after ACL reconstruction?

We hypothesized that both ATT and rTR are strongly correlated to the amount of (Δ)PTS.

## Methods

To answer the research questions, subjects with acute ACL injury were kinematically assessed using in vivo kinematic motion analysis. Dynamic ATT and rTR were measured during level walking, a single-leg hop for distance (SLHD) and a side jump. This study was set up as a multicentre prospective cohort study. Both University Medical Center Groningen (UMCG) and Martini Hospital (Groningen, the Netherlands) included subjects in the study. The study protocol was reviewed and approved by the institutional review board of the UMCG (ID 2015/524). The study was registered in the Dutch Trial Register (NTR: www.trialgregister.nl, registration ID NL7686). From June 2016 to June 2018 all patients diagnosed with ACL injury in one of the two participating hospitals were screened for eligibility to participate in the study. Inclusion criteria were: (1) age 18–35 years, (2) unilateral ACL injury confirmed by physical examination, (3) less than three months post- injury at time of diagnosis, (4) at least six weeks of conservative therapy, (5) intact contralateral knee on physical examination. Exclusion criteria were: (1) any history of fractures, osteotomy, or previous ligament reconstructive surgery in the lower extremities or spine, (2) neurological conditions leading to musculoskeletal disorders, (3) any other musculoskeletal pathology of the lower limbs (i.e. concomitant ligament or meniscal injuries), (4) inability to complete Dutch-language questionnaires.

### Surgical procedure

All subjects underwent anatomic, single-bundle ACL reconstruction using a semitendinosus/gracilis graft. Both tendons were doubled to create a 4-strand graft. For femoral fixation a suspension type fixation was used (Endobutton, Smith&Nephew, London, UK). After pretensioning (60 N), tibial fixation was performed by using a PEEK screw and plug (Biosure PK, Smith&Nephew, London, UK).

### Data collection

The motion data collection was performed at the motion lab of the UMCG’s department of Rehabilitation Medicine. The motion lab consists of a 9 m walkway with two 40 × 60 cm force plates (AMTI; Watertown, MA) embedded in the floor. An 8-camera optoelectronic motion capture system (VICON MX, Vicon Motion Systems Ltd., Oxford, UK) sampling at 100 Hz was used. The position of 22 14 mm spherical markers, distributed on the lower extremities according to Hayes and Davis, was recorded [7]. After static and dynamic calibration, joint centres were calculated using VICON Nexus software v2.8 (VICON MX, Vicon Motion Systems Ltd., Oxford, UK). For the complete procedure and its sensitivity see Keizer and Otten (2020) [19].

All subjects performed three tasks: (1) level walking at a self-selected pace; (2) a single-leg hop for distance (SLHD, maximum forward jump, jumping from and landing on the same leg); and (3) side jump (maximum sideways jump, jumping from and landing on the same leg). All jump trials were performed with hands in free motion and with sport shoes on. To familiarize subjects with the procedure and to make sure the entire foot would land on the force plate, subjects were asked to perform a dry run of the SLHD consisting of three practice trials. The median of the three practice hops was used to determine the starting distance from the force plates. For the side jump, leg length (greater trochanter tip to lateral malleolus tip) was used as starting distance from the centre of the force plates. Trials were included in the analysis when tasks were performed correctly (i.e. stable landing), the entire foot landed on the force plate, and all markers were left in place. Three correct trials were recorded for each leg. ACL-deficient subjects were tested within three months after injury. Approximately 13 months after the first trial, 12 months after ACL reconstruction, the testing procedure was repeated.

### Data processing

The positions of the markers provided data to determine pelvis, femoral, tibial and foot segments. Using VICON Nexus software v2.8 and additional custom MATLAB version 9.7 scripts (The MathWorks Inc., Natick, MA, USA), three dimensional angular displacements and translations in the knee joint were calculated. Data processing and analysis started at initial contact and continued for 200 ms. Initial contact was defined as the moment at which the vertical ground-reaction force (GRF) was > 5% of the body weight. All data were smoothed using the cross-validated quintic spline. Raw 3D marker position data were filtered by using a low pas frequency convolution filter of 10 Hz with zero lag. A maximum gap (temporary absence of marker identification) of 10 frames was accepted to fill in using the software. If a trial contained gaps exceeding 2.5 ms, smoothing of the data could not be performed and trials were discarded. Kinematic variables were quantified and included maximum knee flexion, maximum knee extension, maximum knee valgus, maximum knee varus, maximum anterior tibial translation, range of tibial rotation, and knee flexion moment. Knee flexion moment was calculated from the GRF vector and its lever arm to the center of the knee flexion axis of the stance leg. For quantification of ATT, rTR and knee angles, two coordinate systems were reconstructed in the tested leg using the customized MATLAB script based on the method of Boeth et al. [4] One system was reconstructed in the femoral segment (parent system) and one in the tibial segment (child system). The motion of each coordinate system is consistent with the movement of the respective segment. The ATT was quantified in millimeters using the relative movement of the center of rotation of the tibial coordinate system relative to the center of rotation of the femoral coordinate system in the local tibial coordinate system. The range of tibial rotation was quantified by the angle between the two axes of rotation as outlined by Keizer and Otten [19]. Flexion/extension and varus/valgus angles were obtained using scalar products as in the equations explained by Robertson et al. [26]

### Measurement of PTS

As part of usual care, all subjects underwent magnetic resonance imaging (MRI) of the injured knee to exclude concomitant injury. The images were used to calculate medial and lateral PTS using the circle method as described by Hudek et al. [15] A customized MATLAB script (The MathWorks, Inc., Natick, MA, USA) was used to measure both medial and lateral PTS on MRI. Two independent observers measured both medial and lateral PTS on all MRIs twice, with a minimum two-week interval. To determine intra- and interobserver reliability of the PTS measurements, intraclass correlation coefficients (ICC, 2-way random, absolute agreement) were calculated. Values lower than 0.5 were considered indicative of poor reliability, values between 0.5 and 0.75 indicated moderate reliability, between 0.75 and 0.9 good reliability, and greater than 0.90 excellent reliability [22].

### Statistical analysis

Statistical analysis was performed using SSPS (v 23; IBM Corp, Armonk, NY, USA). A general linear model was used to test for differences between the three trials. Means were calculated for each subject over the three trials to obtain one value for ATT and rTR for each movement. A mean value of medial and lateral PTS from both observers and both measurements was used for analysis.

To assess the correlation between PTS and ATT and between PTS and rTR, Spearman’s correlation coefficients were calculated. This was performed for medial PTS, lateral PTS and ΔPTS. Correlation coefficients were interpreted according to criteria set by Domholdt et al.: 0.00–0.25 represents little if any correlation; 0.26–0.49 weak correlations; 0.50–0.69 moderate; 0.70–0.89 strong; and 0.90–1.00 very strong correlations [11]. To reduce the effect of multiple testing, statistical tests deemed significant if *P* < 0.02.

## Results

A total of 394 subjects were diagnosed with ACL injury and screened for eligibility. Fifty-seven subjects matched the inclusion criteria and were invited to participate in the study. Eleven subjects provided informed consent and were included in the study. The data of one subject was not used for analysis due to the subject’s inability to perform the jumping tasks at the initial session. Six males and four females (N = 10) completed the baseline testing procedures. At follow-up, 12 months after surgery seven subjects remained (N = 7), as one subject had sustained a re-rupture (four months after reconstruction, due to a new trauma) and two subjects were lost to follow-up as they moved away from the Groningen region. The first measurements from the subjects lost to follow up were included when comparing ACL-deficient knees to contralateral ACL-intact knees (N = 10). Patient characteristics and measured PTS values are presented in Table 1.

**Table 1:** Patient Characteristics and PTS values

	Mean (SD)
Age	24 (4.4) years
Total body height	184 (10) cm
Total body weight	81.3 (8.9) kg
Body mass index	24.0 (2.1) kg/m^2^
Injury-to-surgery interval	4.6 months
Medial PTS	- 6.7 (2.5) degrees
Lateral PTS	- 5.7 (2.0) degrees
Δ PTS	- 1.0 (3.5) degrees

ΔPTS = difference between medial PTS and lateral PTS. *PTS* Posterior tibial slope, *SD* Standard deviation

Intraobserver reliability for the medial PTS showed an ICC of 0.82 for observer 1 and 0.83 for observer 2. For the lateral PTS, the ICC for intraobserver reliability was 0.39 for observer 1 and 0.30 for observer 2. Interobserver reliability for the medial PTS demonstrated an ICC of 0.82 and 0.46 for the lateral PTS.

**Fig. 1:**
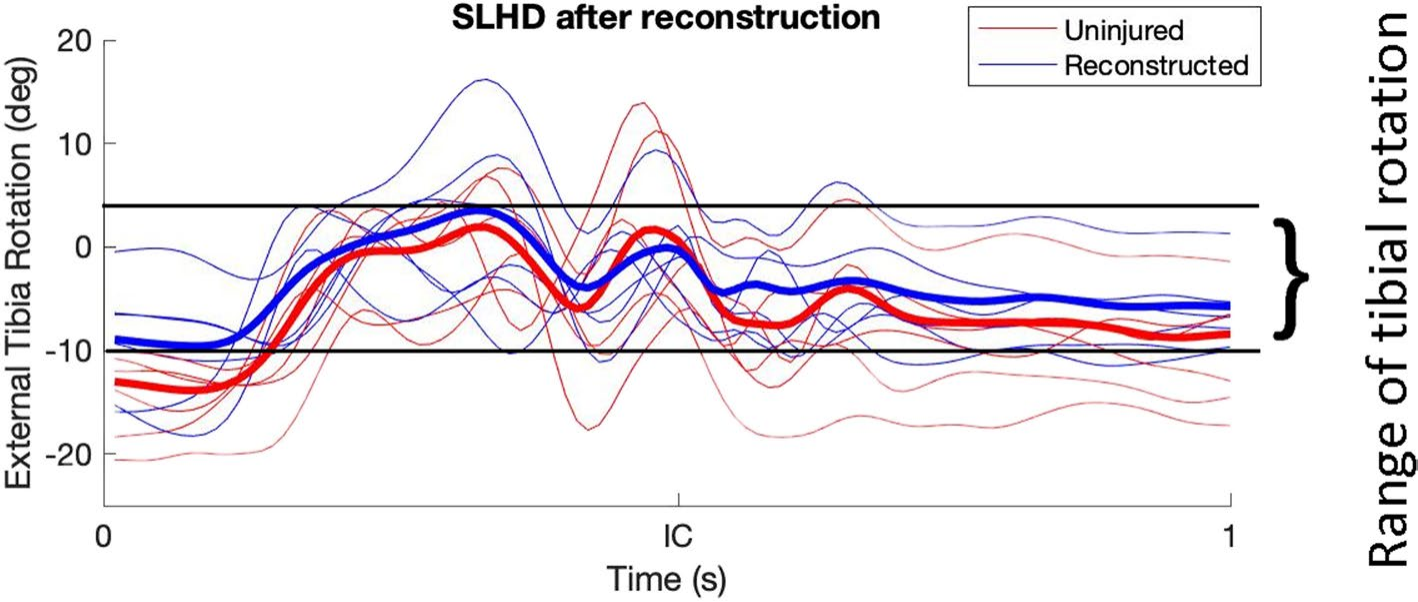
Example of results regarding the range of tibial rotation in both ACL injured knees (red lines) and ACL reconstructed knees (blue lines). The averages are depicted using the bold red and blue line respectively, The solid black lines represent the upper and lower limit of the range of rotation, in this example from the ACL injured knees

The mean values for rTR and ATT during the different movements are displayed in Table 2 for the contralateral ACL-intact, the ACL-deficient and the ACL-reconstructed knees. Compared to the contralateral ACL-intact knees, both the ACL-deficient and the ACL-reconstructed knees showed no significant difference in terms of ATT and rTR. (see Table 2). As an example, Fig. 1 shows a graph containing the results of the rTR during SLHD both before and after reconstruction.

**Table 2:** rTR and ATT during different movements in ACL-deficient, ACL-reconstructed and ACL-intact knees

Kinematic variable	ACL-deficient	ACL-reconstructed	ACL-intact
***Range of tibial rotation (in degrees; mean (SD))***			
Level walking	13.7 (4.1) ^a^(P = 0.15,ns)	14.1 (3.9) ^b^(P = 0.12,ns)	17.3 (6.4)
SLHD	16.9 (3.7) ^a^(P = 0.21,ns)	18.4 (3.4) ^b^(P = 0.64,ns)	19.4 (5.5)
Side jump	16.6 (5.8) ^a^(P = 0.08,ns)	18.3 (4.7) ^b^(P = 0.24,ns)	20.7 (3.6)
***Anterior tibial translation (in mm; mean (SD))***			
Level walking	4.6 (4.8) ^a^(P = 0.13,ns)	4.8 (5.4) ^b^(P = 0.25,ns)	6.6 (3.0)
SLHD	9.3 (5.1) ^a^(P = 0.21,ns)	11.7 (9.2) ^b^(P = 0.60,ns)	13.4 (7.2)
Side jump	6.7 (5.5) ^a^(P = 0.65,ns)	8.8 (7.5) ^b^(P = 0.78,ns)	7.7 (5.8)

^a^ paired t-test results comparing the ACL-deficient knee to the contralateral ACL-intact knee

^b^ paired t-test results comparing the ACL-reconstructed knee to the contralateral ACL-intact knee

*ACL* Anterior cruciate ligament, *SLHD* Single-leg hop for distance, *SD* Standard deviation, *mm* Millimeter, *ns* Non-significant result

The Spearman’s correlation coefficients are displayed in Tables 3 and 4 respectively. Little (if any) to weak correlations were found between medial PTS, lateral PTS and Δ PTS and ATT for ACL-deficient or for ACL-reconstructed knees in all three dynamic tests. Little (if any) to weak correlations were found for ACL-deficient knees between medial PTS, lateral PTS, and ΔPTS and rTR in all three dynamic tests. In ACL-reconstructed knees, these correlations were all moderate-to-strong, except for the correlations between lateral PTS and rTR during level walking and side jump (little (if any) correlation) and medial PTS and rTR during level walking (weak correlation).

**Table 3:** Spearman’s correlation coefficient and significance level for the correlation between ATT and different types of slope

	**Spearman Correlation Coefficient (*ρ*) (Significance level (P))**	
	ACL-deficient	ACL-reconstructed
***ATT and medial PTS***		
Level walking	*ρ* = -0.19 (P = 0.60,ns)	*ρ* = -0.07 (P = 0.88,ns)
SLHD	*ρ* = -0.13 (P = 0.73,ns)	*ρ* = -0.14 (P = 0.76,ns)
Side jump	*ρ* = -0.18 (P = 0.63,ns)	*ρ* = -0.18 (P = 0.70,ns)
***ATT and lateral PTS***		
Level walking	*ρ* = 0.08 (P = 0.83,ns)	*ρ* = 0.29 (P = 0.54,ns)
SLHD	*ρ* = 0.44 (P = 0.20,ns)	*ρ* = -0.11 (P = 0.82,ns)
Side jump	*ρ* = 0.25 (P = 0.49,ns)	*ρ* = 0.18 (P = 0.70,ns)
***ATT and Δ PTS***		
Level walking	*ρ* = -0.26 (P = 0.47,ns)	*ρ* = -0.39 (P = 0.38,ns)
SLHD	*ρ* = -0.47 (P = 0.17,ns)	*ρ* = -0.04 (P = 0.94,ns)
Side Jump	*ρ* = -0.46 (P = 0.19,ns)	*ρ* = -0.43 (P = 0.34,ns)

*ACL* Anterior cruciate ligament, *SLHD* Single-leg hop for distance, *SD* Standard deviation, *PTS* Posterior tibial slope, *ns* Non-significant result

**Table 4:** Spearman’s correlation coefficient and significance level for the correlation between rTR and different types of slope

	**Spearman Correlation Coefficient (*ρ*) (Significance level (P))**	
	ACL-deficient	ACL-reconstructed
***Range of tibial rotation and medial PTS***		
Level walking	*ρ* = -0.21(P = 0.56,ns)	*ρ* = -0.39 (P = 0.38,ns)
SLHD	*ρ* = 0.48 (P = 0.16,ns)	*ρ* = 0.64 (P = 0.12,ns)
Side Jump	*ρ* = 0.44 (P = 0.20,ns)	*ρ* = 0.69 (P = 0.06,ns)
***Range of tibial rotation and lateral PTS***		
Level walking	*ρ* = -0.50 (P = 0.14,ns)	*ρ* = -0.04 (P = 0.94,ns)
SLHD	*ρ* = 0.10 (P = 0.78,ns)	*ρ* = 0.54 (P = 0.22,ns)
Side Jump	*ρ* = 0.08 (P = 0.83,ns)	*ρ* = -0.14 (P = 0.74,ns)
***Range of tibial rotation and ΔPTS***		
Level walking	*ρ* = 0.21 (P = 0.56,ns)	*ρ* = -0.50 (P = 0.25,ns)
SLHD	*ρ* = 0.32 (P = 0.41,ns)	*ρ* = -0.64 (P = 0.12,ns)
Side Jump	*ρ* = 0.37 (P = 0.29,ns)	*ρ* = 0.71 (P = 0.05,ns)

*ACL* Anterior cruciate ligament, *SLHD* Single-leg hop for distance, *SD* Standard deviation, *PTS* Posterior tibial slope, *ns* Non-significant result

It must be noted that the results of the Spearman’s correlation test showed non-significant results, as shown in Tables 3 and 4.

## Discussion

Our study aimed to examine whether PTS is correlated to either ATT or rTR during high-demand activities. The main finding was little (if any) to weak correlation between dynamic ATT and PTS, both before and after ACL reconstruction. By studying subjects using an in vivo motion-capture system, the dynamic forces of the muscles surrounding the knee joint were enabled, in contrast to what happens when measuring passive ATT. The influence of muscle activity may have led to a weak correlation between PTS and dynamic ATT in our study. Earlier studies show a correlation between PTS and ATT in a passive situation, and particularly that an increase in PTS leads to increased passive ATT [8–10, 23, 24, 27, 28, 33, 34]. This previously observed correlation between PTS and passive ATT might be the sole representation of the mechanical interaction between the femur and the tibial slope, as explained by Dejour and Bonnin [10]. Our study suggests that muscular activity enables subjects to compensate for anatomical factors such as PTS by moderating their muscle activation patterns and kinematics when studied during high-demand activities. Dynamic ATT, as measured in our study, is clinically more relevant than passive ATT, as the clinical feeling of giving way is experienced during high-demand activities.

Muscle forces may be able to reduce dynamic ATT in ACL deficiency and after ACL reconstruction. We indeed found that the measured values for both dynamic rTR and ATT seemed lower in ACL-deficient knees and ACL-reconstructed knees compared to their contralateral intact limbs, although this difference was not significant. This may be explained by reduced quadriceps activity of the injured limb, which increases hamstrings-to-quadriceps ratio (HQ ratio). As shown in a 3D computer model by Shelburne et al., reducing quadriceps force can lower ATT in the presence of ACL deficiency [29]. This theory is referred to as the quadriceps avoidance pattern. Moreover, computer models showed that an increase in hamstrings activity, also leading to an increased HQ ratio, likewise reduces the dynamic ATT [29, 31]. Although the theory of altered muscle activation to reduce dynamic ATT is supported by several authors [2, 16, 21, 29], it has been refuted by Keizer et al. [18], who studied healthy subjects with an intact ACL in vivo. In their study, subjects with lax knees on instrumented Lachman displayed less dynamic ATT during SLHD than subjects with lower ATT on instrumented Lachman. Electromyography (EMG) obtained during the SLHD landing showed no clear relation between muscle activity patterns and dynamic ATT, yet less knee flexion was shown by subjects with lax knees. Keizer et al. concluded that landing kinematics may be more relevant than muscle activation in controlling dynamic ATT. Chmielewski et al. found landing kinematics comparable to Keizer et al., i.e. less knee flexion, in subjects with acute ACL injury [5]. In our study these landing kinematics were not seen; no significant difference was observed in maximum knee flexion or knee extension between ACL-intact and ACL-deficient knees.

Several compensation techniques may be successful in reducing dynamic ATT, such as altering landing kinematics or altering muscle activation patterns. A subject’s (biomechanical or anatomical) profile may result in preference for a compensation technique, but most likely it is a complex interplay of many factors. A 3D model fed with material properties, geometrical data, and experimental data (kinematics and EMG data) during dynamic tasks may provide more insight into possible compensation techniques to reduce dynamic ATT. Factors such as self-efficacy, psychological readiness, and subjective knee function may also play an important role. As shown in our earlier work (Zee et al.), psychological readiness and subjective knee function are related to dynamic rTR in ACL deficiency and after ACL reconstruction.

This study is the first to explore a correlation between PTS and dynamic rTR. As with dynamic ATT, little (if any) to weak correlations between dynamic rTR and PTS were observed in ACL deficiency. More specifically, little (if any) to weak correlations were found between dynamic rTR and ΔPTS in ACL deficiency. In acute ACL injury, similarly to the mechanism involved in reducing ATT, diminished hamstring muscle activity has shown to be related to decreased internal rotation of the tibia in ACL-reconstructed subjects [1]. This emphasizes the possibility of the hamstrings influencing rTR, and in doing so, counteracting the influence of PTS on rTR in acute ACL deficiency. However, one year after ACL reconstruction we have observed moderate-to-strong correlations between rTR and PTS. This may indicate that the previously hypothesized compensation mechanisms fail to compensate for rotatory laxity in the long run. Taking these factors into account, caution should be exercised with highly invasive procedures such as an anterior closing-wedge osteotomy of the tibia. Theoretically, a tibial osteotomy will influence the biomechanical interaction between passive ATT and PTS but neglects the (powerful) influence of muscle activation. Ultimately, the correlation between PTS and ATT may be corrected by muscle activation, but this may not be the case for the correlation between PTS and rTR. Hence the possibilities of an alternative osteotomy technique to correct for tibial rotation, for instance an anteromedial opening wedge, may be explored.

### Limitations and future research

This study has several limitations. The narrow inclusion and exclusion criteria were mainly responsible for the small sample size—for instance, subjects with concomitant injury were excluded. Injury to the menisci and anterolateral structures of the knee are known to influence the amount of tibial rotation [20]. By including subjects with concomitant injury, the results could have been biased. Although concomitant injury is a common feature in the general population, we regard our results as an accurate representation of the biomechanics involved in solitary ACL deficiency. The limited sample size is mainly responsible for the non-significant result of the correlation tests. However correlation coefficients are more relevant when interpreting Spearman’s test as opposed to significance levels. Nonetheless the results our study urge the need for future studies with more subjects to confirm the correlations found. Our study did not include electromyography (EMG) measurements to support our theory. In future research it would be interesting to incorporate the use of EMG to evaluate muscle activation patterns during SLHD in ACL deficiency and after ACLR.

The average medial PTS in our population was -6.7° (95% CI -4.9; -8.5), and in the lateral compartment -5.7° (95% CI -4.3; -7.1). It must be noted that interobserver and intraobserver agreement was poorer for lateral PTS compared to medial PTS. Still, our observed PTS values are comparable to previous studies. In a systematic review and meta-analysis by Wordeman et al., average lateral PTS in ACL-injured subjects was between -1.8 (± 3.2) and -11.5 (± 3.54) degrees [35]. Average medial PTS in ACL-injured subjects was between + 1.8 (± 3.5) and -12.1(± 3.3) degrees.[35].

We cannot state whether the aforementioned compensation mechanisms are able to limit ATT in subjects with higher levels of PTS. Dejour et al. report a significant increase of passive ATT with PTS > 12°[8], Li et al. report increased passive ATT with PTS of 10°and Webb et al. report increased risk of ACL injury and graft failure with PTS > 12° [23, 34]. Observed PTS did not reach these values in our population. It would be of interest to additionally investigate the relation between PTS and ATT during in vivo motion. The ΔPTS variable is theoretically interesting to explore further with respect to tibial rotation.

## Conclusions

In contrast to passive ATT, which is significantly correlated to PTS, little (if any) to weak correlations were found between dynamic ATT and PTS. A compensation mechanism seems to be able to correct for the anatomical influence of PTS on dynamic ATT during high-demand tasks. Moderate-to-strong correlations between PTS and dynamic rTR were found one year after ACL reconstruction. These findings warrant prudence in the use of a pure anterior closing-wedge osteotomy in ACL reconstruction; the effect of an anteromedial opening wedge on dynamic ATT and rTR may be further explored.

## Data Availability

All data is available from the corresponding author upon reasonable request.

## Abbreviations

ACL: Anterior cruciate ligament
ATT: Anterior tibial translation
EMG: Electromyography
PTS: Posterior tibial slope
rTR: Range of tibial rotation
SLHD: Single-leg hop for distance
SD: Standard deviation
